# A randomized, multi-center, prospective study comparing best medical treatment versus best medical treatment plus renal artery stenting in patients with hemodynamically relevant atherosclerotic renal artery stenosis (RADAR) – one-year results of a pre-maturely terminated study

**DOI:** 10.1186/s13063-017-2126-x

**Published:** 2017-08-14

**Authors:** Thomas Zeller, Hans Krankenberg, Andrejs Erglis, Erwin Blessing, Torsten Fuss, Dierk Scheinert, Ralf Weser, Beatrix B. Doerr, Wilfrid D. Yollo, Joerg Radermacher, for the RADAR Investigators

**Affiliations:** 1 Department of Angiology, Universitäts-Herzzentrum Freiburg-Bad Krozingen, Südring 15, 79189 Bad Krozingen, Germany; 2 Department of Angiology, Asklepios Klinik Hamburg, Hamburg, Germany; 3 Latvian Center of Cardiology, P. Stradins Clinical University Hospital, Riga, Latvia; 4 Department of Internal Medicine, SRH Klinikum Karlsbad-Langensteinbach, Karlsbad, Germany; 5 Zentralklinikum Suhl, Suhl, Germany; 6 Department Internal Medicine, Elblandkliniken, Radebeul, Germany; 7 Department of Internal, Neurological and Dermatological Medicine, Division of Interventional Angiology, Universitätsklinikum Leipzig, Leipig, Germany; 8 Abt. Kardiologie und Angiologie, Herzzentrum Coswig, Coswig, Anhalt Germany; 9 Coriuvar Clinical Research & Medical Writing, Moosburg, Germany; 10 Statistical Programming Pool (S2P), Yaounde, Cameroon; 11 Center for Internal Medicine/Nephrology, Klinikum I, Johannes Wesling Klinikum Minden, Minden, Germany

**Keywords:** Renal artery stenosis, Renal artery stenting, Best medical treatment, Best medical therapy, Renal function, Hypertension

## Abstract

**Background:**

The indications for conservative “best medical treatment” (BMT) versus additional renal artery stenting are a matter of ongoing debate. The RADAR study aimed to evaluate the impact of percutaneous renal artery stenting on the impaired renal function in patients with hemodynamically significant atherosclerotic renal artery stenosis (RAS).

**Methods:**

RADAR is an international, prospective, randomized (1:1) controlled study comparing BMT alone versus BMT plus renal artery stenting in patients with duplex sonographic hemodynamically relevant RAS. Follow-up assessments were at 2, 6, and 12 months and at 3 years. The primary endpoint was change in estimated glomerular filtration rate (eGFR) at 12 months.

**Results:**

Due to slow enrollment, RADAR was terminated early after inclusion of 86 of the scheduled 300 patients (28.7%). Change in eGFR between baseline and 12 months was 4.3 ± 15.4 ml/min/1.73 m^2^ (stent group) and 3.0 ± 14.9 ml/min/1.73 m^2^ (BMT group), *p* > 0.999. Clinical event rates were low with a 12-month composite of cardiac death, stroke, myocardial infarction, and hospitalization for congestive heart failure of 2.9% in the stent and 5.3% in the BMT group, *p* = 0.526, and a 3-year composite of 14.8% and 12.0%, *p* = 0.982. At 3 years, target vessel (re-)vascularization occurred in one patient (3.0%) in the stent group and in 8 patients (29.4%) in the BMT group.

**Conclusion:**

In RADAR, outcomes of renal artery stenting were similar to BMT. These results have to be interpreted with the caveat that the study did not reach its statistically based sample size.

**Trial registration:**

Clinicaltrials.gov, NCT00640406. Registered on 17 March 2008.

**Electronic supplementary material:**

The online version of this article (doi:10.1186/s13063-017-2126-x) contains supplementary material, which is available to authorized users.

## Background

Atherosclerosis accounts for approximately 90% of cases of renal artery stenosis (RAS). It is a progressive disease [1], with more than half of the patients exhibiting an increasing degree of stenosis within 5 years after diagnosis [2], and one out of five patients with critical stenosis suffers renal atrophy and renal failure during this period [3].

RAS may be treated conservatively with “best medical treatment” (BMT), surgically, or by endovascular interventions using balloon angioplasty and stenting [4]. The indications for endovascular treatment are a matter of ongoing debate. Curing hypertension by means of angioplasty rarely occurs, although the number of antihypertensive medications usually can be reduced after successful treatment. While observational cohort studies have shown beneficial effects of stenting compared to BMT, randomized controlled trials (CORAL, ASTRAL, and STAR) have not. The outcomes in the latter were due to overly liberal inclusion criteria, e.g. inclusion of hemodynamically insignificant lesions with a stenosis diameter < 70% [4, 5].

The RADAR study therefore aimed to evaluate the clinical impact of percutaneous renal artery stenting on renal function measured by estimated glomerular filtration rate (eGFR) in patients with hemodynamically significant atherosclerotic RAS, based on duplex ultrasonographic patient screening [6].

## Methods

### Study design and population

RADAR is an international, multicenter, randomized controlled study comparing BMT with BMT plus renal artery stenting (stent group) in patients with hemodynamically relevant atherosclerotic RAS. The design of the trial has been described previously [6]. In brief, the study was conducted in 15 enrolling centers in Europe and Brazil. Patients were randomized to receive renal artery revascularization using the Dynamic renal stent system (Biotronik AG, Buelach, Switzerland) plus BMT or BMT alone.

Subjects were eligible if they presented with hemodynamically relevant de novo unilateral or bilateral atherosclerotic RAS based on the following criteria: in unilateral RAS, the difference in intrarenal resistance index (dRI) > 0.05 and in bilateral disease an acceleration time > 0.10 s. Additional inclusion criteria were eGFR > 10 ml/min, hypertension and/or renal dysfunction, and renal reference vessel diameter ≥ 4.0 mm and < 7.0 mm based on visual estimation. Main exclusion criteria were renal atrophy, prior revascularization of the target lesion, causes of RAS other than atherosclerosis, and chronic renal replacement therapy. The trial is registered at Clinicaltrials.gov, NCT00640406 and the clinical investigation plan is available upon request.

Randomization was performed in a 1:1 fashion using a centralized randomization system. Data were collected at baseline/intervention, at 2-month, 6-month, and 12-month follow-up, and after 3 years.

The RADAR study was conducted according to the current version of the Declaration of Helsinki, International Harmonization Conference (ICH) good clinical practice (GCP) principles and ISO 14155:2003, and local regulations as applicable. The study was approved at the respective ethic committees and all subjects provided informed consent. To ensure high-data quality, study subjects were monitored on site. All safety endpoints were adjudicated by an independent clinical events committee.

### Study device

The Dynamic renal stent is a tubular, balloon-expandable stent sculpted by laser from a single tube of L-605 cobalt-chromium alloy. The stent surface is fully coated with a layer of amorphous hydrogen-rich silicon carbide. This material has semiconducting properties and reduces the conversion of fibrinogen to fibrin [7], the adhesion and activation of blood platelets and leucocytes [8], and the release of potentially allergenic ions. The stent delivery system is based on a rapid-exchange balloon catheter. It is compatible with guiding catheters with a minimal inner diameter of 0.070″ (1.78 mm, 6F) or introducer sheaths of 4–5 F.

### Procedures

The intervention for patients randomized to stenting had to be performed following the locally established standard procedure. Antiplatelet therapy before the intervention was acetyl salicylic acid (ASA) 100 mg/day and clopidogrel 300 mg/day each on one day before the intervention and on the day of intervention (clopidogrel could be replaced by administering ticlopidine 500 mg/day one day prior to the procedure). Alternatively a bolus dose of 500 mg of ASA and clopidogrel 600 mg had to be administered on the day of the procedure. Prior to the intervention, a bolus dose of 2500–10,000 IU of heparin had to be given. Further concomitant medication was left at the discretion of the investigator. Dual antiplatelet therapy had to be administered for at least 4 weeks and ASA infinitely.

All patients had to be treated according to individualized BMT. BMT was defined as optimal drug therapy for hypertension control, low salt diet, and treatment with ASA and lipid-lowering therapy (e.g. statins). Individualized BMT had to be applied using the locally established standards or knowledge in order to reach the following limits: LDL, ≤ 100 mg/dL; blood pressure, ≤ 125/80 mmHg for 24 h-blood pressure; and HbA1c, ≤ 6.5%. Patients not reaching these target limits were kept in the study, but effort had to be made to apply the appropriate medication to meet the limits.

### Endpoints and definitions

Endpoints have been reported in detail previously [6]. The primary endpoint was change in eGFR at 12 months. Secondary endpoints were: technical success, defined as successful access and deployment of the device with appropriate lesion coverage, stent positioning and patency determined by angiography; acute procedural success, defined as residual diameter stenosis < 30% by quantitative angiography; procedural success, defined as successful lesion crossing and stent positioning without the occurrence of a serious adverse event up to the moment the introducer sheath was removed; change in eGFR at 2 and 6 months; further change in renal function; ratio of average resistance index (RI); degree of restenosis; change in kidney length (pole to pole); left ventricular mass index (LVMI); systolic and diastolic blood pressure; number of anti-hypertensive drugs; New York Heart Association (NYHA) classification; and quality of life. Secondary endpoints up to 3 years were clinical events such as major adverse cardiac and cerebrovascular events (MACCE), a composite of cardiac death, stroke, myocardial infarction, and hospitalization for congestive heart failure; renal or cardiac death; stroke; myocardial infarction; hospitalization for congestive heart failure; progressive renal insufficiency, i.e. need for dialysis; need for permanent replacement therapy; and need for target vessel revascularization (TVR) or target lesion (re-)vascularization (TLR).

### Statistical analysis

The sample size was based on the primary endpoint. Based on published data and assuming 15–20% of patients with bilateral stenosis, ΔeGFR for the stent group was calculated as ≈ 5.5 ml/min. For the BMT group, a difference of − 1.0 ml/min was estimated. Considering a dropout rate of approximately 20%, and a stenting rate of 20% of patients being initially randomized to the BMT group, a total of 300 patients had to be enrolled (150 patients per group) [6].

The analysis is based on the intention-to-treat (ITT) population (i.e. subjects were analyzed in the groups to which they were randomized, and no subjects were excluded from the analysis). Patients who received a bailout stenting before 6-month follow up were considered protocol violators and counted in the ITT population, but not for the per-protocol analysis. According to the study protocol, patients in the BMT group who received bailout stenting after 6 months were originally censored at the time of stent placement and excluded from 12-month analysis, but based upon peer review, we included those patients in the clinical outcome analysis. Descriptive statistics are presented for the purpose of this report. Quantitative variables are reported as mean and standard deviation and minimum to maximum respective median and interquartile ranges when appropriate. Qualitative variables are reported as frequencies and percentages. Survival analysis of clinical events was performed using the Kaplan-Meier estimator (95% CI for the estimates are also presented).

For comparison between treatments the following tests were used: Fisher’s exact (for comparison of categorical data, e.g. degree of renal stenosis, blood pressure categories), the chi-squared test (for baseline categorical variables), Student’s *t* test (for comparison of mean within the groups, e.g. age), and the log-rank test (for survival analysis). The Wilcoxon signed rank test was used for inter-individual comparison (e.g. primary endpoint and duplex sonography). All tests were two-sided with a 0.05 significant level. Poisson regression was performed in a post-hoc analysis. All statistical analyses were carried out using SAS version 9.3 or a later version (SAS Institute Inc. Cary, NC, USA).

## Results

**Fig. 1:**
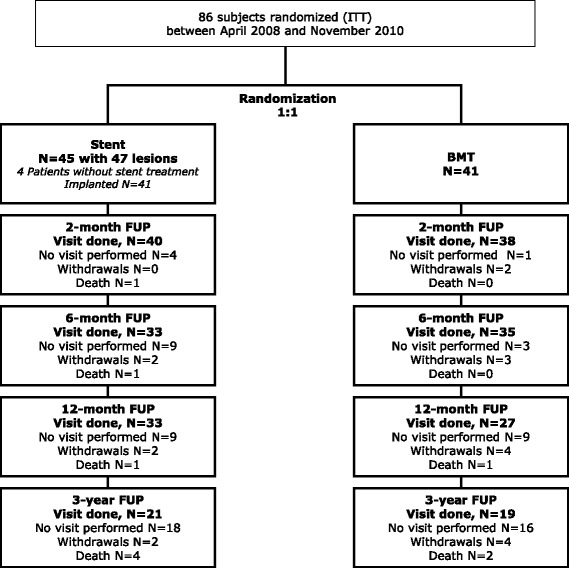
Patient flow chart. There were 3 patients in the best medical treatment (*BMT*) group who received bailout stenting before 6 months, 3 patients received it between 6 and 12 months, and 4 patients received it beyond 12 months. *ITT* intention to treat, *FUP* follow up

Patients were enrolled from April 2008 to November 2010. As several attempts to increase the enrollment rate failed and the average enrollment rate was fewer than 3 patients per month, enrollment was terminated when only 86 out of the targeted 300 patients (28.7%) were included. Of the 45 patients enrolled in the stent group, 4 did not undergo a study procedure. Of the remaining 41 patients, 6 (14.6%) had treatment of two lesions. Not all centers participated in the 3-year follow up and hence data are available on only a limited number of patients as shown in Fig. 1.

Baseline characteristics are displayed in Table 1. About half of the patients had coronary artery disease and about one third of the patients had peripheral artery disease or diabetes mellitus. There was no statistically significant difference amongst the groups. Mean angiographic stenosis diameter was 80.2 ± 9.4%. All stents were successfully delivered and technical success was obtained with nearly all devices (Table 2).

**Table 1:** Baseline patient characteristics

	Stent group *N* = 45	BMT group *N* = 41
Male gender	32 (71.1)	28 (68.3)
Age, years	67.2 ± 8.4, 44–88	64.8 ± 12.1, 40–84
Smoking history^a^	25 (55.6)	20 (48.8)
Diabetes mellitus^b^	14 (31.1)	16 (39.0)
Coronary disease	25 (55.6)	20 (48.8)
Peripheral artery disease	15 (33.3)	16 (39.0)
Congestive heart failure	16 (35.6)	14 (34.1)

Data are displayed as number (percentage) or mean ± SD, minimum–maximum. *BMT* best medical treatment. ^a^Data unavailable on two patients in each group. ^b^Data unavailable on one patient in the BMT group

**Table 2:** Procedural characteristics of the stent group

	Stent group *N* = 47
Lesion length, mm	10.4 ± 3.9, 2–25
Ostial lesion	44 (93.6)
Stenosis diameter, %	80.2 ± 9.4, 48–100
Stent diameter,	
5.0 mm/6.0 mm/7.0 mm	8 (17.0)/28 (59.6)/11 (23.4)
Residual stenosis after stenting, %	2.3 ± 4.9, 0–25
Lesion completely covered by stent	45 (95.7)
Technical success	45 (95.7)
Acute procedural success	44 (93.6)
Procedural success	45 (95.7)

Data are displayed as number (percentage) or mean ± SD, minimum–maximum. Technical success was defined as successful access and deployment of the device with appropriate lesion coverage, stent positioning, and patency determined by angiography. Acute procedural success was defined as residual diameter stenosis < 30% assessed by quantitative angiography. Procedural success was defined as successful lesion crossing and stent positioning without the occurrence of a serious adverse event up to the moment the introducer sheath was removed

Change in eGFR between baseline and 12 months was 4.3 ± 15.4 ml/min/1.73 m^2^ (95% CI, − 1.7, 10.3) ranging from − 26 to 41 ml/min/1.73 m^2^ in the stent group and 3.0 ± 14.9 ml/min/1.73 m^2^ (95% CI, − 3.5, 9.4) ranging from − 34 to 22 ml/min/1.73 m^2^ in the BMT group, *p* > 0.999 (Table 3). In both treatment arms the change between baseline and 12 months was not significant (*p* = 0.277 in the stent group and *p* = 0.197 in the BMT group). Compared to baseline, in the stent group versus the BMT group there was improvement in 10 patients (35.7%) versus 10 patients (43.5%), stabilization in 13 patients (46.4%) versus 9 patients (17.4%), and failure in 5 patients (17.9%) versus 4 patients (17.4%), respectively (*p* = 0.839).

**Table 3:** Mean eGFR at baseline versus follow-up (ml/min/1.73 m^2^)

	ITT unpaired data			ITT paired data			Per-protocol paired data		
	Stent	BMT	*P* value	Stent	BMT	*P* value	Stent	BMT	*P* value
Baseline	52.6 ± 22.1	55.9 ± 21.0	0.589	57.0 ± 20.8	60.8 ± 21.3	0.725	57.0 ± 20.8	61.4 ± 21.7	0.653
2 month	55.4 ± 20.1	55.9 ± 22.2	0.940	57.4 ± 20.9	58.8 ± 21.2	0.967	57.4 ± 20.9	58.7 ± 21.7	0.983
6 month	59.1 ± 26.6	56.7 ± 24.5	0.800	57.7 ± 22.2	60.1 ± 22.9	0.877	57.7 ± 22.2	60.2 ± 23.5	0.872
12 month	59.8 ± 22.4	62.3 ± 21.6	0.767	60.1 ± 24.1	63.1 ± 21.8	0.649	60.1 ± 24.1	63.3 ± 22.3	0.700
Change baseline–2 months	0.5 ± 9.5	−2.2 ± 10.1	0.277	0.4 ± 9.5	− 2.0 ± 10.3	0.844	0.4 ± 9.5	− 2.8 ± 9.9	0.660
Change baseline − 6 months	1.3 ± 12.5	− 0.6 ± 14.5	0.549	0.7 ± 12.0	− 0.7 ± 15.3	0.656	0.7 ± 12.0	− 1.2 ± 15.5	0.507
Change baseline–12 months	4.3 ± 15.4	3.0 ± 14.9	> 0.999	3.1 ± 15.3	2.3 ± 14.9	0.812	3.1 ± 15.3	1.9 ± 15.1	0.906

The ITT population includes the three BMT patients receiving bailout stenting prior to 6 months and includes patients receiving bailout stenting after 6 months until they received stenting. The per-protocol analysis excludes patients with bailout stenting prior to 6 months and 2 patients in the stent group who received a non-study stent. Unpaired data were available at baseline, 2, 6, and 12 months in 70, 67, 61, and 54 patients, respectively and data were available on change at 2, 6, and 12 months compared to baseline in 61, 58, and 51 patients, respectively. Intention-to-treat (ITT) paired data were available in 28 patients in the stent group and in 23 patients in the BMT group, for the per protocol analysis in 26 and 21 patients, respectively. Unpaired data at respective time points refer to the data available and change refers to change in the data based on paired datasets. Paired data means that datasets were available at all time points. *BMT* best medical treatment, *eGFR* estimated glomerular filtration rate

Renal ultrasound data are displayed in Table 4 (paired data in Additional file 1: Table S1). All patients receiving a stent were free of 70% restenosis at follow up except for one. Median change in the LVMI was − 3.0 g/m^2^ in the stent group and + 3.0 g/m^2^ in the BMT group, *p* = 0.285.

**Table 4:** Renal duplex sonography

	Baseline		6 months		12 months	
	Stent^a^	BMT^a^	Stent^a^	BMT^a^	Stent^a^	BMT^a^
Renal aortic ratio	4.6 ± 2.0	5.0 ± 2.3	1.7 ± 0.9 (< 0.001)^b^	4.5 ± 2.6 (0.462)^b^	1.5 ± 0.6 (< 0.001)^b^	3.0 ± 1.5 (0.004)^b^
*P* value	0.527		< 0.001		0.003	
Maximal systolic flow, cm/s	317 ± 94	360 ± 102	147 ± 51 (< 0.001)^b^	355 ± 133 (0.178)^b^	138 ± 44 (< 0.001)^b^	301 ± 117 (0.125) ^b^
*P* value	0.061		< 0.001		< 0.001	
Renal resistive index	0.69 ± 0.09	0.65 ± 0.12	0.73 ± 0.10 (0.005)^b^	0.63 ± 0.12 (0.594)^b^	0.73 ± 0.08 (< 0.001)^b^	0.63 ± 0.13 (0.438)^b^
*P* value	0.240		< 0.001		0.006	
Kidney length, pole to pole, mm	99 ± 11	104 ± 14	105 ± 14 (0.044)^b^	98 ± 11 (0.066)^b^	105 ± 14 (0.071)^b^	99 ± 13 (0.090)^b^
*P* value	0.280		0.031		0.138	
Acceleration time, s	0.12 ± 0.06	0.11 ± 0.07	0.07 ± 0.05 (0.003)^b^	0.10 ± 0.07 (0.502)^b^	0.10 ± 0.06 (0.249)^b^	0.16 ± 0.06 (0.500)^b^
*P* value	0.925		0.186		0.026	
Degree of stenosis						
> 70%	47 (97.9)	38 (97.4)	0 (0.0)	31 (91.2)	1 (3.1)	15 (79.0)
< 70%	1 (2.1)	1 (2.6)	37 (100.0)	3 (8.8)	31 (96.9)	4 (21.1)
*P* value	> 0.999		< 0.001		< 0.001	

Data are shown as number (percentage) or mean ± SD. Intention-to-treat population, unpaired data. *BMT* best medical treatment

^a^Some assessments could be performed in all patients

^b^ *P* value for analysis of change in time in pairs compared to baseline

**Fig. 2:**
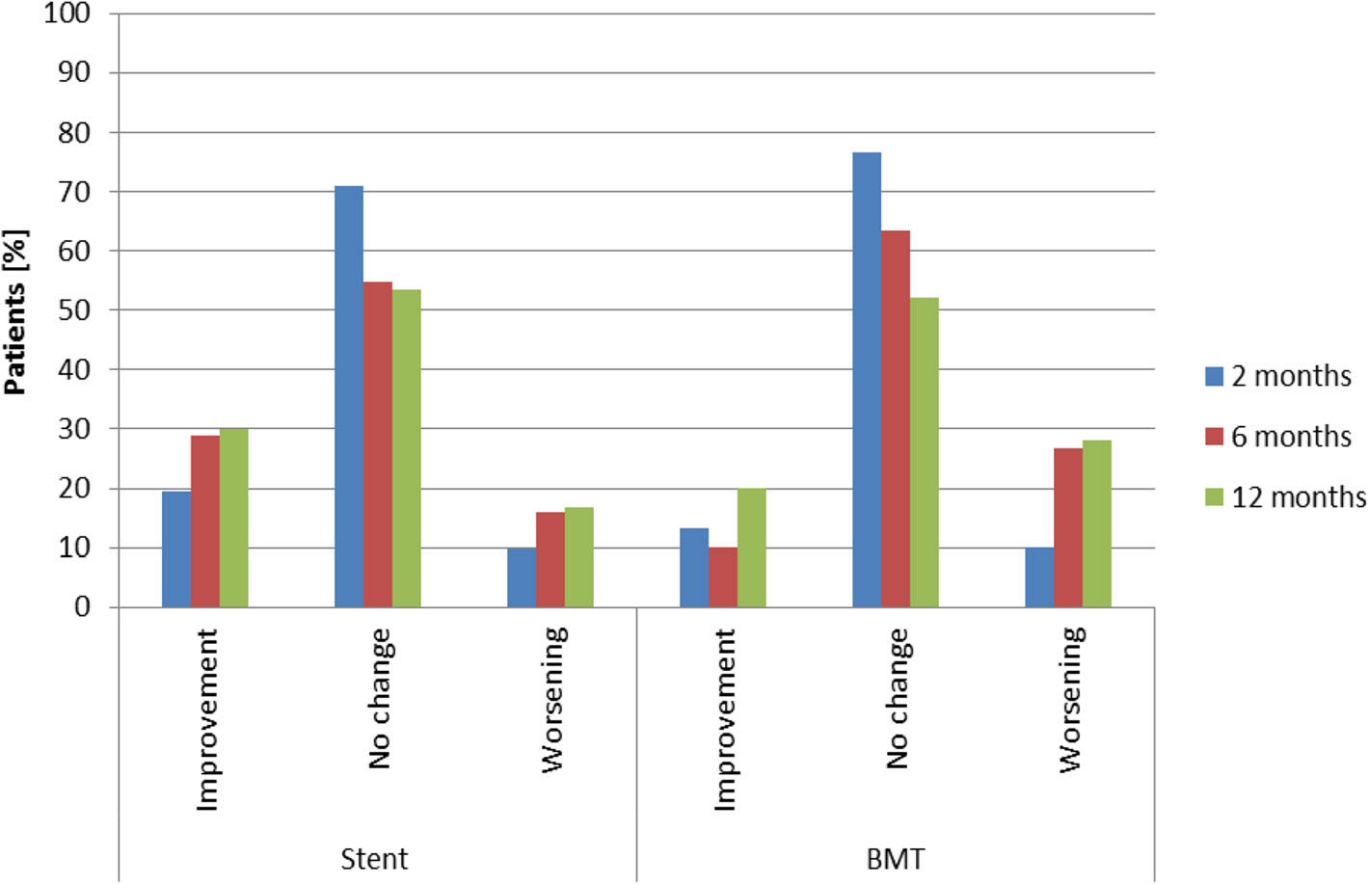
Percentage of patients with change in antihypertensive medication compared to baseline. There was no statistical significant difference between the groups. *BMT* best medical treatment

At baseline, less than 10% of patients in each group had moderate hypertension, none had severe hypertension, and 39% had isolated hypertension (Table 5). As measured per 24-hour blood pressure analysis at 12 months, mean systolic blood pressure improved by 7 mmHg in the stent group compared to 5 mmHg in the BMT group (*p* = 0.980) (Additional file 1: Table S2). Patients in the stent group received 2.4 ± 1.4 antihypertensive medications and patients in the BMT group received 2.8 ± 2.0 antihypertensive medications, on average at 12 months (*p* = 0.523). Compared to baseline, improvement in medication was observed in 30% of the stent group and 20% of the BMT group (Fig. 2).

**Table 5:** Blood pressure at baseline and follow up

	Baseline		6 months		12 months	
	Stent	BMT	Stent	BMT	Stent	BMT
Optimal	7 (18.4)	3 (9.1)	8 (25.8)	9 (29.0)	7 (25.9)	8 (30.8)
Normal	5 (13.2)	8 (24.2)	7 (22.6)	6 (19.4)	8 (29.6)	8 (30.8)
High normal	6 (15.8)	4 (12.1)	7 (22.6)	9 (29.0)	5 (18.5)	6 (23.1)
Grade 1 HTN (mild)	2 (5.3)	2 (6.1)	0 (0.0)	2 (6.5)	2 (7.4)	1 (3.8)
Grade 2 HTN (moderate)	3 (7.9)	3 (9.1)	0 (0.0)	0 (0.0)	0 (0.0)	0 (0.0)
Grade 3 HTN (severe)	0 (0.0)	0 (0.0)	0 (0.0)	0 (0.0)	0 (0.0)	0 (0.0)
Isolated systolic HTN	15 (39.5)	13 (39.4)	9 (29.0)	5 (16.1)	5 (18.5)	3 (11.5)
*P* value	0.785		0.549		0.946	

Data are displayed as number (percentage). *BMT* best medical treatment, *HTN* hypertension

Two patients died prior to 12 months. One patient in the stent group died on day 7 due to procedure-related multi-organ failure following surgical revision of retroperitoneal bleeding resulting from an unrecognized rupture of the renal artery, and one patient in the BMT group died on day 235 due to multi-organ failure as a result of initial pneumonia. One patient in the stent group had target lesion revascularization (TLR) and target vessel revascularization (TVR) at the 12-month visit on day 346 post procedure, and in the BMT group four patients with six lesions were stented within 12 months. In the centers that participated in the 3-year follow up, four additional stents were inserted in patients in the BMT group (Table 6). The results of poisson regression analysis are displayed in Additional file 1: Table S3.

**Table 6:** Kaplan-Meier time-to-event estimates of clinical outcomes

	12 months			3 years		
	Stent *N* (%) (95% CI)	BMT *N* (%) (95% CI)	*P* value	Stent *N* (%) (95% CI)	BMT *N* (%) (95% CI)	*P* value
MACCE^a^	1 (2.9) (0.4, 19.1)	2 (5.3) (1.3, 19.5)	0.526	4 (14.8) (5.8, 35.1)	3 (12.0) (3.5, 36.8)	0.982
Death	1 (2.3) (0.3, 15.4)	1 (3.1) (0.4, 20.2)	0.955	4 (13.8) (5.3, 33.3)	2 (9.2) (2.2, 34.1	0.639
Renal death	1 (2.2) (0.3, 14.7)	0 (0.0)	1.000	1 (2.2) (0.3, 14.7)	0 (0.0)	1.00
Cardiac death	0 (0.0)	0 (0.0)	> 0.999	2 (7.9) (2.0, 28.1)	0 (0.0) (0.0, 0.0)	0.495
Stroke	1 (2.9) (0.4, 19.1)	1 (2.7) (0.4, 17.7)	0.966	1 (2.9) (0.4, 19.1)	2 (9.7) (2.2, 37.3)	0.447
Myocardial infarction	0 (0.0)	0 (0.0)	> 0.999	1 (4.3) (0.6, 27.1)	0 (0.0) (0.0, 0.0)	> 0.999
Hospitalization for congestive heart failure	0 (0.0)	1 (2.6) (0.4, 16.8)	0.477	1 (4.2) (0.6, 26.1)	1 (2.6) (0.4, 16.8)	0.855
Progressive renal insufficiency (i.e. need for dialysis)	1 (2.4) (0.3, 16.1)	1 (2.7) (0.4, 17.7)	0.978	1 (2.4) (0.3, 16.1)	1 (2.7) (0.4, 17.7)	0.978
Permanent renal replacement therapy	1 (2.4) (0.3, 16.1)	0 (0.0)	> 0.999	1 (2.4) (0.3, 16.1)	0 (0.0)	> 0.999
Target vessel (re)vascularization	1 (3.0) (0.4, 19.6)	4 (11.0) (4.3, 26.8)	0.142	1 (3.0) (0.4, 19.6)	8 (29.4) (15.4, 51.6)	0.008
Target lesion (re)vascularization	1 (3.0) (0.4, 19.6)	4 (11.0) (4.3, 26.8)	0.142	1 (3.0) (0.4, 19.6)	8 (29.4) (15.4, 51.6)	0.008

Data are displayed as number (percentage) (95% CI). *BMT* best medical treatment, *MACCE* major adverse cardiac and cerebrovascular event

^a^Composite of cardiac death, stroke, myocardial infarction and hospitalization for congestive heart failure

Antihypertensive therapy, NYHA classification, Short form-12 (SF-12) quality of life outcomes and laboratory data are provided in Additional file 1: Tables S4–S7.

## Discussion

Following the negative outcome of the ASTRAL study [9], despite best efforts applied in screening patients, participating investigators faced difficulties in attracting eligible subjects for enrollment. Moreover, patients were already transferred to stenting when visiting the site and therefore were reluctant to be randomized when one arm would receive treatment with medication only. Due to the very slow enrollment rate, the sponsor in consultation with the study Coordinating Clinical Investigator initiated several efforts to increase enrollment rates, but success was limited. As a result, enrollment was terminated on 1 December 2010 when only 28.7% of the total cohort was enrolled. Thus, sufficient statistical power was never achieved and conclusions on clinical and functional outcomes should be taken with caution.

Attributed to duplex ultrasonographic screening, which is considered to be most selective for detecting hemodynamically relevant RAS [10, 11], baseline angiographic renal artery diameter stenosis in the interventional cohort (80.2 ± 9.4%) was highest in RADAR when compared to other randomized trials [4, 5, 9].

For the primary endpoint of change in the eGFR, the results were similar between groups but with a small difference in favor of the stent group. While values in the stent group were comparable to those predicted in the sample size calculation, values in the BMT group were better than expected (increase of 3.0 ml/min/1.73 m^2^ versus estimated decrease of − 1.0 ml/min/1.73 m^2^).

As assessed by renal duplex sonography, stenting was highly effective in reducing restenosis. Only one lesion in the stent group had restenosis with a stenosis diameter > 70%, whereas ten lesions in eight patients in the BMT group needed bailout stenting (three prior to 6 months, three between 6 and 12 months, and four after 12 months). As expected the renal resistive index increased in the stent group, while the maximal systolic flow declined. However, aside of the change in perfusion, the renal resistive index can also be influenced by systemic hemodynamics and the presence of microvascular abnormalities [12]. Eventually, the intervention may also have showered the renal parenchyma and converted a macrovascular stenosis into a parenchymal stenosis. At 6 months, the kidney size, measured as pole-to-pole length, increased in the stent group (mean length of 99 mm at baseline compared to 105 mm at follow up, *p* = 0.044), but decreased in the BMT group (104 mm at baseline compared to 98 mm at follow up, *p* = 0.066), which might be reflective of reactivating hibernating parenchyma in the kidneys following restoration of blood supply in some patients, whereas progressive fibrosis might cause the trend towards shrinking kidney size in those under conservative treatment.

As shown in former studies [13, 14], LVMI was reduced numerically in the stent cohort whereas a mild increase was found in the BMT cohort, yet the difference amongst the group was not statistically significant (*p* = 0.285). This is most likely due to the fact that significant RAS causes activation of the renin-angiotensin-aldosterone system with aldosterone as the strongest promoter of left ventricular hypertrophy [15, 16].

Mean blood pressure was reduced in both groups. Outcomes were similar between the groups, but the stent group reached the goal with administration of fewer antihypertensive medications. When interpreting the results, the relatively mild form of hypertension at baseline in most patients has to be considered. Segrest et al. [17] reported that reductions in blood pressure > 10 mmHg are only observed in studies with a mean systolic blood pressure > 160 mmHg, RAS > 85% and > 2.6 medications. Similarly, in a patient-level pooled analysis of 901 patients, Weinberg et al. [18] found that systolic blood pressure > 150 mmHg was positively related with reduction in blood pressure.

Clinical event rates were low. Up to 3 years, approximately one third of patients in the BMT group received a stent in the renal artery. Considering that nearly all lesions in the stent group were ostial lesions, it is encouraging that TLR only occurred in one patient in this group.

### Limitations

When assessing the results, the following confounders have to be considered. Only six patients in the stent group had two lesions treated. If there is unilateral disease, the contralateral kidney can compensate for the diseased kidney, hence masking the effect of treatment. Patients with more tissue at risk are naturally more likely to respond to treatment as do patients with bilateral disease or only one kidney in place. Furthermore, nearly one third of the patients had diabetes mellitus at baseline, a factor that might have had an impact on renal function [4].

In general, the evidence from randomized trials comparing stenting with BMT is still insufficient. The STAR trial was underpowered [19], and the ASTRAL and CORAL trials have generated much debate and controversy, but no finality. Neither of the trials demonstrated greater benefit of stenting compared to BMT, in contrast to large interventional cohort studies and meta-analysis. The reason for the lack of evidence is likely associated with major flaws in the study design and study conduct (e.g. inclusion of patients with stenosis < 50%), which invalidated the results and which are discussed in detail in recent reviews [4, 20]. Also, the studies did not evaluate patients who did not respond to BMT and those who were not eligible for inclusion in the study [21]. The RADAR study adds a piece to this puzzle. However, RADAR did enroll less than one third of its statistically calculated sample size. Aside of the small number of patients, our trial has the limitation that it enrolled at least some patients with reduced risk as it allowed for controlled hypertension, and some patients did not meet all the inclusion and exclusion criteria. The inclusion of patients not meeting all eligibility criteria might have been induced by the low enrollment rates; however, we acknowledge that it is also an expression of poor study conduct.

Meanwhile, the latest SCAI expert consensus published in 2014 [21] reports stenting as appropriate treatment in patients with flash pulmonary edema or acute coronary syndrome with severe hypertension, in patients with resistant hypertension, defined as failure of maximally tolerated doses of at least three antihypertensive medications, or in patients with ischemic nephropathy and chronic kidney disease with eGFR < 45 cc/min and global renal ischemia; only a minority of patients in the RADAR study met those criteria. Furthermore, it is questionable whether the study hypothesis using the eGFR was appropriate.

There may also have been selection bias, as severely diseased patients who were transferred for renal artery stenting might not have been enrolled in RADAR, and instead only those patients in whom it was unclear which treatment option would be beneficial were selected. Furthermore, the follow-up compliance was low, a phenomenon that is often seen in trials with slow recruitment with only a few patients per site.

Further trials should focus: (1) on exploring parameters that could predict beneficial outcomes, e.g. renal frame count, as common parameters such as diameter stenosis correlate poorly with outcomes [5, 17] and (2) on the potential benefit of renal artery stenting in specific patient subsets such as patients with heart failure [21, 22] or patients such as those defined as “maybe appropriate care” by the recent SCAI consensus, e.g. patients with unilateral RAS and eGFR < 45 cc/min [21]. A collaboration program with transferring physicians should be initiated to identify those patients before they are transferred to stenting.

## Conclusions

In RADAR, the outcomes of renal artery stenting were similar to BMT, but a non-negligible number of patients in the BMT group underwent renal stenting during follow up. Outcomes have to be interpreted taking into account several study limitations and with the caveat that the study enrolled less than one third of the statistically based required sample size.

## Additional file

Additional file 1: Table S1. Renal duplex sonography, paired data. **Table S2** Blood pressure (mmHg) at baseline and follow up. **Table S3.** Clinical outcomes per poisson regression. **Table S4.** Antihypertensive therapy. **Table S5.** NYHA classification. **Table S6.** Significance levels for improvements in quality of life (SF-12 questionnaire). **Table S7.** Laboratory data at baseline and follow up. (DOC 191 kb)

## Abbreviations

ASA: Acetyl salicylic acid
BMT: Best medical treatment
dRI: Difference in intrarenal resistance index
eGFR: Estimated glomerular filtration rate
ITT: Intention to treat
LVMI: Left ventricular mass index
MACCE: Major adverse cardiac and cerebrovascular events
NYHA: New York Heart Association
RAS: Renal artery stenosis
RI: Resistance index
TLR: Target lesion revascularization
TVR: Target vessel revascularization
