# Supplementary material for: A randomized, multi-center, prospective study comparing best medical treatment versus best medical treatment plus renal artery stenting in patients with hemodynamically relevant atherosclerotic renal artery stenosis (RADAR) – one-year results of a pre-maturely terminated study
Source: Trials. 2017 Aug 14;18:380. doi: 10.1186/s13063-017-2126-x (PMC5556660; doi:10.1186/s13063-017-2126-x)
Supplement: Additional file 1: Table S1. — Renal duplex sonography, paired data. Table S2 Blood pressure (mmHg) at baseline and follow up. Table S3. Clinical outcomes per poisson regression. Table S4. Antihypertensive therapy. Table S5. NYHA classification. Table S6. Significance levels for improvements in quality of life (SF-12 questionnaire). Table S7. Laboratory data at baseline and follow up. (DOC 191 kb) [file 13063_2017_2126_MOESM1_ESM.doc]

**Table S1: Renal duplex sonography, paired data**

1. ITT population

| ***ITT population - paired data*** | | | | | | |
| --- | --- | --- | --- | --- | --- | --- |
|  | **Baseline** | | **6 months** | | **12 months** | |
|  | **Stent^a^**  **N=38** | **BMT^a^** | **Stent^a^** | **BMT^a^** | **Stent^a^** | **BMT^a^** |
| **Renal Aortic Ratio^a^** | 5.1±1.8 | 5.4±1.9 | 1.7±0.8  (<0.001)^b^ | 6.1±2.7  (0.320) ^b^ | 1.5±0.5  (<0.001)^b^ | 3.6±1.1  (0.004)^b^ |
| *p-value* | 0.719 | | <0.001 | | <0.001 | |
| **Maximal systolic flow, cm/s** | 332±68 | 358±90 | 133±29  (<0.001)^b^ | 341±82  (0.375)^b^ | 119±30  (<0.001)^b^ | 301±125  (0.125)^b^ |
| *p-value* | 0.608 | | <0.001 | | 0.032 | |
| **Renal Resistive Index** | 0.67±0.09 | 0.61±0.11 | 0.73±0.06  (<0.001)^b^ | 0.59±0.11  (0.280)^b^ | 0.73±0.08  (<0.001)^b^ | 0.62±0.13  (0.641)^b^ |
| *p-value* | 0.038 | | <0.001 | | 0.004 | |
| **Kidney pole to pole length, mm** | 98±12 | 101±15 | 105±14  (0.010)^b^ | 99±12  (0.338)^b^ | 106±14  (0.031)^b^ | 97±14  (0.145)^b^ |
| *p-value* | 0.578 | | 0.318 | | 0.173 | |
| **Acceleration time, s** | 0.09±0.09 | 0.10±0.05 | 0.06±0.05  (0.022)^b^ | 0.11±0.1  (0.125)^b^ | 0.08±0.05  (0.249)^b^ | 0.17±0.07  (0.375)^b^ |
| *p-value* | 0.823 | | 0.309 | | 0.052 | |
| **Degree of stenosis**  >70%  <70% | 30 (96.8)  1 (3.2) | 14 (93.3)  1 (6.7) | 0 (0.0)  31 (100.0) | 14 (93.3)  1 (6.7) | 1 (3.2)  30 (96.8) | 13 (86.7)  2 (13.3) |
| *p-value* | >0.999 | | <0.001 | | <0.001 | |

Data are shown as n (%), or mean±SD. BMT-best medical therapy.

^a^not all assessments could be done in all patients, ^b^p-value for change-in-time analysis in pairs compared to baseline

1. Per protocol population

| ***Per protocol population - paired data*** | | | | | | |
| --- | --- | --- | --- | --- | --- | --- |
|  | **Baseline** | | **6 months** | | **12 months** | |
|  | **Stent^a^**  **N=38** | **BMT^a^** | **Stent^a^** | **BMT^a^** | **Stent^a^** | **BMT^a^** |
| **Renal Aortic Ratio** | 5.1± | 5.4±1.9 | 1.7±0.8  (<0.001)^b^ | 6.1±2.7  (0.320)^b^ | 1.5±0.5  (<0.001)^b^ | 3.6±1.1  (0.004)^b^ |
| *p-value* | 0.719 | | <0.001 | | <0.001 | |
| **Maximal systolic flow, cm/s** | 332±69 | 358±90 | 133±29  (<0.001)^b^ | 341±82  (0.375)^b^ | 119±30  (<0.001)^b^ | 301±125  (0.125)^b^ |
| *p-value* | 0.601 | | <0.001 | | 0.032 | |
| **Renal Resistive Index** | 0.67±0.09 | 0.60±0.11 | 0.73±0.06  (<0.001)^b^ | 0.57±0.10  (0.110)^b^ | 0.73±0.08  (<0.001)^b^ | 0.61±0.13  (0.814)^b^ |
| *p-value* | 0.028 | | <0.001 | | 0.001 | |
| **Kidney pole to pole length, mm** | 98±12 | 100±15 | 105±14  (0.010)^b^ | 97±10  (0.238)^b^ | 106±14  (0.031)^b^ | 95±15  (0.156)^b^ |
| *p-value* | 0.788 | | 0.179 | | 0.227 | |
| **Acceleration time** | 0.10±0.05 | 0.09±0.09- | 0.06±0.05  (0.022)^b^ | 0.11±0.10  (0.125)^b^ | 0.08±0.05  (0.249)^b^ | 0.17±0.07  (0.375)^b^ |
| *p-value* | 0.823 | | 0.309 | | 0.052 | |
| **Degree of stenosis**  >70%  <70% | 30 (96.8)  1 (3.2) | 13 (92.9)  1 (7.2) | 0 (0.0)  31 (100.0) | 14 (100.0)  0 (0.0) | 1 (3.2)  30 (96.8) | 13 (92.9)  1 (7.2) |
| *p-value* | 0.530 | | <0.001 | | <0.001 | |

Data are shown as n (%), or mean±SD. BMT-best medical therapy.

^a^not all assessments could be done in all patients, ^b^p-value for change-in-time analysis in pairs compared to baseline

The per protocol analysis excludes patients with bailout stenting prior to 6 months and 2 patients in the stent group which received a non-study stent.

**Table S2: Blood pressure [mmHg] at baseline and follow-up**

|  | **Baseline** | | **6 months** | | **12 months** | |
| --- | --- | --- | --- | --- | --- | --- |
|  | **Stent** | **BMT** | **Stent** | **BMT** | **Stent** | **BMT** |
| **Supine systolic BP** | 146±20  120–210 | 150±25  90–216 | 146±27  100–235 | 140±18  110–190 | 142±26  110–244 | 148± 4  99–200 |
| *p-value* | 0.312 | | 0.307 | | 0.160 | |
| **Supine diastolic BP** | 80±13  50–120 | 82±12  64–110 | 80±15  56–120 | 80±15  56–120 | 82±13  55–113 | 82±12  64–111 |
| *p-value* | 0.741 | | 0.897 | | 0.814 | |
| **24 hours BP systolic** | 139±18  97–172 | 140±18  98–170 | 130±14  106–160 | 129±17  105–173 | 129±13  107–157 | 128±16  98–171 |
| *p-value* | 0.705 | | 0.887 | | 0.696 | |
| **24-hours BP diastolic** | 78±14  52–106 | 74±16  51–120 | 72±9  56–89 | 72±11  53–94 | 74±11  56–101 | 71±10  54–93 |
| *p-value* | 0.246 | | 0.970 | | 0.299 | |
| **Change in 24-hours BPs systolic** ^a^ | - | - | -5  (-21 – 2)  (0.003)^b^ | -10  (-21 – 4)  (0.017)^b^ | -7  (-21 – 2)  (0.023)^b^ | -5  (-24 – 4)  (0.088)^b^ |
| *p-value* | - | | 0.862 | | 0.980 | |
| **Change in 24-hours BPs diastolic** ^a^ | - | - | -3  (-12 – 1)  (0.076)^b^ | -3  (-7 – 1)  (0.008)^b^ | -4  (-10 – 6)  (0.404)^b^ | -4  (-8 – 5)  (0.234)^b^ |
| *p-value* | NA | | 0.601 | | 0.850 | |

Data are displayed as mean ± SD, Min–Max; or median, (Quartile 1 – Quartile 3). BMT-best medical therapy, BP-blood pressure HTN-hypertension

^a^ Comparison with baseline values. Only paired data was considered, ^b^ p-value for change in time analyses

**Poisson estimates of clinical outcomes**

For estimation of the rate r of event per year (events-year), we considered the simple Poisson regression model:

log (r) = B*randomization group + log (duration of follow up in days/365) + e

Which is equivalent to **r = exp (B*randomization group + log (duration of follow up in days/365) + e)**

Where **B** is the vector of regression coefficients, **log** is the link function, **log (duration of follow up in days/365)** is an **offset** in the model equation. The offset account for different observation periods for different subjects

We divided the follow up period in days by 365 to receive the rate by years (PE°) instead of days.

In case the model do not converges or we have not enough observation for the modelling NA for not applicable is reported.

Table S3 Clinical outcomes per poisson regression

|  | **12 months** | | **3 years** | |
| --- | --- | --- | --- | --- |
|  | **Stent** | **BMT** | **Stent** | **BMT** |
| **MACCE*** | 1 (1.6) [0.2,11.2] | 2 (4.8) [1.2,19.3] | 4 (0.6) [0.2,1.6] | 3 (1.1) [0.3,3.3] |
| **Death** | NA | 1 (0.7) [0.2,2.6] | 4 (0.5) [0.2,1.5] | 2 (0.7) [0.2,2.6] |
| **Renal death** | NA | NA | NA | NA |
| **Cardiac death** | NA | NA | 2 (0.5) [0.1,2.2] | NA |
| **Stroke** | 1 (1.6) [0.2,11.2] | 1 (3.3) [0.5,23.3] | 1 (1.6) [0.2,11.2] | 2 (0.7) [0.2,3.0] |
| **Myocardial infarction** | NA | NA | 1 (0.4) [0.1,2.9] | NA |
| **Hospitalization for congestive heart failure** | NA | 1 (9.1) [1.3,64.8] | 1 (0.4) [0.1,3.1] | 1 (9.1) [1.3,64.8] |
| **Progressive renal insufficiency (i.e. need for dialysis)** | 1 (14.6) [2.1,103.6] | 1 (5.7) [0.8,40.5] | 1 (14.6) [2.1,103.6] | 1 (5.7) [0.8,40.5] |
| **Target vessel (re)vascularization** | 1 (1.0) [0.1,7.4] | 4 (1.1) [0.5,2.2] | 1 (1.0) [0.1,7.4] | 8 (1.1) [0.5,2.2] |
| **Target lesion (re)vascularization** | 1 (1.0) [0.1,7.4] | 4 (1.1) [0.5,2.2] | 1 (1.0) [0.1,7.4] | 8 (1.1) [0.5,2.2] |

Data are displayed as n (PE°) [95% CI]. *Composite of cardiac death, stroke, myocardial infarction and hospitalization for congestive heart failure. MACCE-major adverse cardiac and cerebrovascular events, NA-not applicable.

**Table S4: Antihypertensive therapy**

|  | **Baseline** | | **2 months** | | **6 months** | | **12 months** | |
| --- | --- | --- | --- | --- | --- | --- | --- | --- |
|  | **Stent** | **BMT** | **Stent** | **BMT** | **Stent** | **BMT** |  |  |
| **Mean number of medication** | 2.4±1.2  1–5 | 2.7±1.9  0–8 | 2.3±1.2  0–5 | 2.7±1.7  1–8 | 2.4±1.3  0–6 | 2.8±1.7  1–8 | 2.4±1.4  0–6 | 2.8±2.0  0–8 |
| *p-value* | 0.964 | | 0.642 | | 0.335 | | 0.286 | |
| **Difference in mean number of medications^a^** | - | - | -0.2±0.7  -2–1  (0.202)^b^ | 0.0±1.0  -2–4  (>0.999)^b^ | -0.1±1.1  -2–3  (0.502)^b^ | 0.2±1.1  -3–3  (0.326)^b^ | -0.1±1.0  -2–3  (0.459)^b^ | 0.0±1.3  -4–2  (0.877)^b^ |
| *p-value* | - | | 0.634 | | 0.1000 | | 0.520 | |
| **Change in medications^a^**  Worsening  No change  Improvement | - | - | 3 (9.7)  22 (71.0)  6 (19.4) | 3 (10.0)  23 (76.7)  4 (13.3) | 5 (16.1)  17 (54.8)  9 (29.0) | 8 (26.7)  19 (63.4)  3 (10.0) | 5 (16.7)  16 (53.3)  9 (30.0) | 7 (28.0)  13 (52.0)  5 (20.0) |
| *p-value* | - | | 0.918 | | 0.172 | | 0.397 | |

Data are displayed as mean ± SD, Min–Max or n (%). BMT-best medical treatment

^a^ Comparison with baseline values. Only paired data was considered

^b^ p-value for change in time analyses

**Table S5: NYHA classification**

|  | **Baseline** | | **2 months** | | **6 months** | | **12 months** | |
| --- | --- | --- | --- | --- | --- | --- | --- | --- |
|  | **Stent** | **BMT** | **Stent** | **BMT** | **Stent** | **BMT** | **Stent** | **BMT** |
| NYHA class  I  II  III  IV | 6 (37.5)  6 (37.5)  3 (18.8)  1 (6.3) | 5 (35.7)  7 (50.0)  2 (14.3)  0 (0.0) | 5 (41.7)  6 (50.0)  1 (8.3)  0 (0.0) | 3 (21.4)  9 (64.3)  2 (14.3)  0 (0.0) | 4 (26.7)  10 (66.7)  1 (6.7)  0 (0.0) | 4 (36.4)  7 (63.6)  0 (0.0)  0 (0.0) | 6 (60.0)  3 (30.0)  1 (10.0)  0 (0.0) | 4 (50.0)  4 (50.0)  0 (0.0)  0 (0.0) |
| *p-value* | > 0.999 | | 0.527 | | > 0.999 | | 0.798 | |

Data are displayed as n (%). BMT-best medical treatment; NYHA-New York Heart Association

.

**Table S6: Significance levels of Quality of Life improvements (SF-12 questionnaire)**

|  | **Baseline vs 6 months** | | **Baseline vs 12 months** | |
| --- | --- | --- | --- | --- |
|  | **Stent** | **BMT** | **Stent** | **BMT** |
| **Physical functioning** | 0.102 | 0.724 | **0.006** | **0.039** |
| **Role Physical** | 0.114 | 0.895 | 0.260 | 0.875 |
| **Bodily Pain** | 0.954 | 0.281 | 0.132 | 0.461 |
| **General Health** | **0.014** | 0.382 | **0.036** | **0.031** |
| **Vitality** | 0.056 | 0.112 | 0.083 | 0.811 |
| **Social Function** | 0.799 | 0.946 | 0.640 | 0.640 |
| **Role Emotional** | 0.454 | 0.959 | **0.013** | 0.484 |
| **Mental Health** | **0.042** | 0.422 | 0.052 | 0.116 |
| **Physical Summary Measure** | 0.208 | 0.452 | 0.243 | 0.134 |
| **Mental Summary Measure** | 0.366 | 0.452 | **0.036** | 0.518 |

BMT-best medical treatment

The SF-12 V2.0 (Norm Based Scoring, US Score) was used as quality of life assessment at baseline, 6 and 12 months follow-up.

In the table above, the significance level between baseline and 6 months and baseline and 12 months for both groups are displayed (all p-values were calculated with Wilcoxon Signed Rank test).

Significance in improvement over 12 months could be shown for Physical Functioning in the Stent group with p= 0.006 and in the BMT group with p= 0.039. For Vitality the stent group was close to significance at 6 and 12 months. General Health improved significantly at 6 months in the stent group and at 12 months in both groups. Role Emotional and Mental Health improved significantly for the stent group only. The QoL composite Mental changed significantly in the stent group with p= 0.036 over twelve months. In summary the stent group showed significantly more improvements over 12 months than the BMT group furthermore improvement (not only significant ones) in all categories were seen in both groups over time.

**Table S7: Laboratory parameters at baseline and follow-up**

| Data are displayed as median, (Quartile 1 - Quartile 3) | **Baseline** | | **2 months** | | **6 months** | | | **12 months** | | |
| --- | --- | --- | --- | --- | --- | --- | --- | --- | --- | --- |
|  | **Stent** | **BMT** | **Stent** | **BMT** | **Stent** | **BMT** | |  |  | |
| **C-reactive protein** [mg/dl] | 1.7  (0.5 - 5.8) | 1.5  (0.2 - 5.6) | 1.7  (0.3 - 4.1) | 0.7  (0.2 - 2.6) | 1.4  (0.3 - 4.9) | 1.0  (0.2 - 5.0) | | 1.1  (0.3 - 4.6) | 0.2  (0.2 - 2.0) | |
| *p-value* | 0.361 | | 0.239 | | 0.526 | | | 0.050 | | |
| **Creatinine**  [mg/dl] | 1.20  (1.01 - 1.35) | 1.10  (0.98 - 1.44) | 1.11  (0.99 - 1.35) | 1.10  (1.00 - 1.40) | 1.08  (0.98 - 1.27) | 1.10  (0.91 - 1.30) | | 1.10  (1.00 - 1.30) | 1.11  (0.90 - 1.27) | |
| *p-value* | 0.568 | | 0.991 | | 0.643 | | | 0.797 | | |
| **BNP**  [pg/ml] | 134  (57 - 408) | 167  (22 - 317) | 106  (46 - 263) | 189  (37 - 278) | 115  (59 - 291) | 100  (29 - 301) | | 116  (59 - 199) | 126  (43 - 250) | |
| *p-value* | 0.379 | | 0.929 | | 0.412 | | | 0.821 | | |
| **HbA1c**  [%] | 5.8  (5.6 - 6.1) | 6.0  (5.5 - 6.7) | 5.8  (5.6 - 6.2) | 5.9  (5.55 - 6.75) | 5.9  (5.6 - 6.6) | | 6  (5.7 - 6.7) | 5.9  (5.6 - 6.5) | | 5.9  (5.5 - 6.45) |
|  | 0.565 | | 0.372 | | 0.748 | | | 0.878 | | |
| **LDL**  [mg/dl] | 101  (80 - 128) | 97  (78 - 128) | 96  (80 - 125) | 86  (64 - 104) | 102  (76 - 123) | | 95  (64 - 118) | 97  (84 - 116) | | 95  (70 - 128) |
| *p-value* | 0.901 | | 0.082 | | 0.308 | | | 0.741 | | |

BNP-brain natriuretic peptid, Hb-hemoglobin, LDL-low density lipoprotein

At baseline and follow-up, the median HbA1c was below the targeted threshold of 6.5% and the median LDL was around the targeted threshold of 100 mg/dl.
